# Advances in the functions of CTRP6 in the development and progression of the malignancy

**DOI:** 10.3389/fgene.2022.985077

**Published:** 2022-10-12

**Authors:** Bo Hu, Xiaolan Qian, Ping Qian, Guangtao Xu, Xin Jin, Deqing Chen, Long Xu, Jie Tang, Wenjing Wu, Wanlu Li, Jin Zhang

**Affiliations:** ^1^ Department of Pathology and Municipal Key-Innovative Discipline of Molecular Diagnostics, Jiaxing Hospital of Traditional Chinese Medicine, Jiaxing University, Jiaxing, China; ^2^ Forensic and Pathology Laboratory, Department of Pathology, Institute of Forensic Science, Jiaxing University, Jiaxing, China; ^3^ College of Biological, Chemical Sciences and Engineering, Jiaxing University, Jiaxing, China

**Keywords:** C1q/TNF-related protein 6, lung cancer, hepatocellular carcinoma, gastric cancer, oral squamous cell carcinoma, bladder cancer, renal cancer

## Abstract

CTRP6, a member of the C1q/TNF-related protein (CTRP) family, has gained increasing scientific interest because of its regulatory role in tumor progression. Previous studies have shown that CTRP6 is closely involved in regulating various pathophysiological processes, including glucose and lipid metabolism, cell proliferation, apoptosis, and inflammation. To date, CTRP6 has been identified as related to eight different malignancies, including lung cancer, oral cancer, gastric cancer, colon cancer, liver cancer, bladder cancer, renal cancer, and ovarian cancer. CTRP6 is reported to be associated with tumor progression by activating a series of related signal networks. This review article mainly discusses the biochemistry and pleiotropic pathophysiological functions of CTRP6 as a new molecular mediator in carcinogenesis, hoping that the information summarized herein could make a modest contribution to the development of novel cancer treatments in the future.

## Introduction

Cancer is a major public health problem worldwide, with incidences increasing yearly (Siegel et al., 2021). In recent years, there has been remarkable progress in the knowledge about the molecular and cellular mechanisms that mediate carcinogenesis. Nevertheless, many problems concerning cancer prevention and treatment remain to be answered (Tsimberidou et al., 2020). In addition, many currently available treatment methods cannot achieve expected satisfactory therapeutic outcomes due to heterogeneity of tumor cells in gene expression, metabolic activity, proliferation, and potential metastasis (Zou and Wang, 2019). Therefore, it is necessary to find and select personalized precise methods for the sake of improving the diagnosis and treatment of malignant tumors.

Obesity has become a more prevalent public health problem and is considered one of the main cancer risk factors (Avgerinos et al., 2019). Obesity has been linked to the initiation and progression of many types of carcinoma (De Pergola and Silvestris, 2013). Recent data suggest that obesity-induced cancer could be triggered by chronic inflammation in adipose tissues, which will produce local genotoxic stress leading to the initiation of malignancies (Lengyel et al., 2018). Obesity, as a chronic inflammatory state of over nutrition, activates the signaling pathway of cell growth factors, thereby increasing the risk of tumor transformation. In the process of cancer initiation and development, mitogen-activated protein kinase (MAPK), Janus kinase (JAK)/signal transducers and activators of transcription (STAT), Akt and phosphatidylinositol 3-kinase (PI3K) signaling pathways are frequently altered (Hopkins et al., 2016). Fatty acids and other metabolic adipocytokines secreted by adipocytes can reduce the immune function of obese patients and facilitate tumor progression (Spyrou et al., 2018). Among these insulin, steroid hormones, cytokines, adipocytokines, leptin, and adiponectin are the most thoroughly studied in tumor development (Vansaun, 2013). In addition, adiponectin is reported to be strongly associated with different types of tumor growth and can enhance tumor aggressiveness (Di Zazzo et al., 2019), suggesting that the crosstalk between fat tissue and tumor cells depends on the effects of adiponectin and its homologs.

Complement 1q tumor necrosis factor-related proteins (CTRPs) are a protein family, acting as adipokines and sharing the similar structure and physiological function of adiponectin in cellular progression (Wong et al., 2004). CTRPs are involved in several biological processes, including chronic inflammation (Jung and Jung, 2021), fibrosis (Zhao et al., 2020), apoptosis (Zhang and He, 2019), autoimmunity (Omeka et al., 2019), cell proliferation and differentiation (Schäffler and Buechler, 2012; Wu et al., 2017). In recent years, the role of CTRP members in carcinogenesis has attracted more attention and interest from researchers (Li et al., 2011; Li et al., 2017; Thanasupawat et al., 2018). Among these, CTRP3, CTRP4, CTRP6, and CTRP8 have been reported to be strongly associated with several malignant tumors, like osteosarcoma (Akiyama et al., 2009), hepatocellular carcinoma (Wan et al., 2019), glioblastoma (Glogowska et al., 2022), and other malignancies (Kong et al., 2021). CTRPs are also involved in the carcinogenesis of multiple tumors and may be considered biomarkers or therapeutic targets (Klonisch et al., 2017). The purpose of this review is to compile the latest literature on CTRP6 related different types of tumor development and progression and discuss recently proposed understanding mechanisms of CTRP6 in tumor progression, including tumor cell proliferation and metastasis, in an attempt to provide the latest insights into the role of CTRP6 in cancer prevention and treatment.

## General structure and function of CTRP6

**FIGURE 1:**
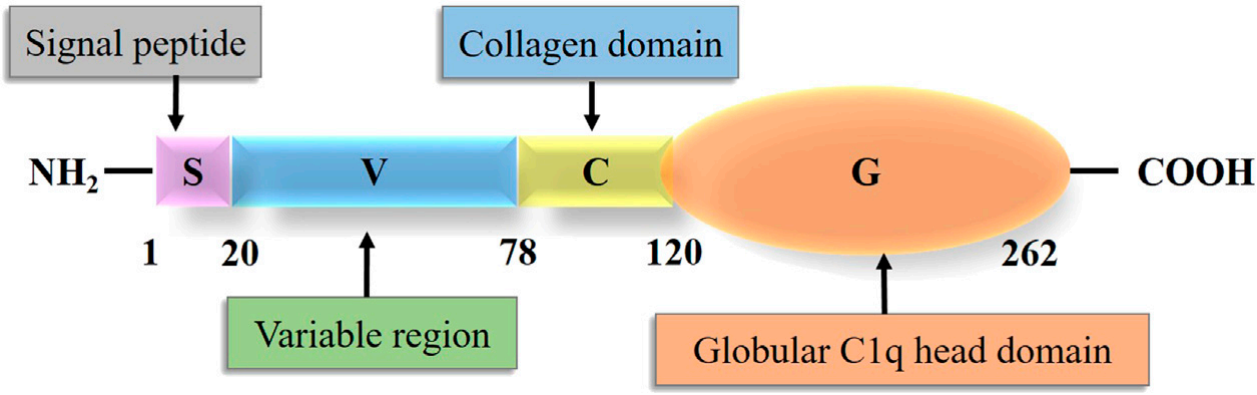
Structure of Complement 1q tumor necrosis factor-related protein 6.

The term CTRP was originally proposed by Wong et al. (2004) to describe a new secretory protein family. CTRPs are cloned according to the sequence homology between CTRPs and adiponectin (Seldin et al., 2014). The CTRP family is composed of 16 members, including CTRP1-9, 9B (Peterson et al., 2009), and 10–15 (Seldin et al., 2014). CTRP6, with a molecular weight of 29 kDa, consists of four domains: N-terminal signal peptide domain, short variable region domain, collagenous domain with various lengths of Gly-X-Y repeats, and C-terminal globular C1q domain (Schäffler and Buechler, 2012). Schematic structure of CTRP6 and the number of amino acid in each domain is shown in the following result (Figure 1). The number of amino acids in each domain is 20, 58, 42, and 142, respectively (Wong et al., 2004). The chromosome location of human CTRP6 is 22q13.1. The collagenous domain has 14 Gly-X-Y (X and Y refer to any amino acid) repeats. The 3D structure of the spherical C1q domain is almost the same as that of the C-terminal region of tumor necrosis factor (TNF) homologous domain (THD), which is a typical feature of TNF family members (Wong et al., 2004). The globular domain of mCTRP6 shares 34% homology with the amino acid sequence of adiponectin (Wong et al., 2004). The C-terminal globular domain is considered a functional domain that may interact with other proteins or receptors.

Increasing numbers of studies have demonstrated that CTRP family members, especially CTRP6, play an integral role in numerous biological processes including inflammation (Lahav et al., 2021), cell proliferation (Dong et al., 2018), apoptosis (Qu et al., 2021), fibrosis (Lei et al., 2015), sclerosis (Zhang et al., 2019), and carcinogenesis (Kong et al., 2021). CTRP6 is reported to be involved in various insulin resistance diseases (Lei et al., 2017), like type 2 diabetes (Wang et al., 2018), obesity (Liao et al., 2021), and diabetic nephropathy (Xu et al., 2020). CTRP6 also shows a close relationship with related cardiac vascular diseases, like cardiac fibrosis (Lei et al., 2015), cardiotoxicity (Zheng et al., 2019), and atherosclerosis (Liu et al., 2021). CTRP6, as an endogenous complement regulator, is highly expressed in the serum of patients with rheumatoid arthritis (Murayama et al., 2015). Recently, the role of CTRP6 in carcinogenesis has aroused increasing interest and attention. The role of CTRP6 in various tumor types has been summarized (Table 1 **)**. CTRP6 is reported to function as a tumor-promoting regulator in several malignancies by promoting tumor cell survival and anti-apoptosis (Kong et al., 2021). It has also been demonstrated to enhance the migration and invasion of cancer cells. However, the mechanism of CTRP6 in tumor initiation and development remains unclear.

**TABLE 1:** CTRP6 expression in various tumor types.

PMID	Types of tumors	Expression in tumor	Relationship with prognosis	Function	Related pathways	Refs.
31292940	Lung adenocarcinoma	Increased	Poor prognosis	Promote cell proliferation, migration, invasion, and inhibit apoptosis	MEK/ERK cascades related MAPK signaling pathway	Han et al. (2019)
33269376						Zhang and Feng (2021)
21508531	Hepatocellular carcinoma	Increased	Not mentioned	Accelerate tumor neovascularization, promote cell proliferation, migration, invasion and inhibit apoptosis	Akt signaling pathway	Wan et al. (2019)
31111552						Takeuchi et al. (2011)
30431096	Gastric cancer	Increased	Favorable prognosis	(Controversial) Promote cell proliferation, migration, invasion, and regulate cell cycle arrest, and inhibit apoptosis; not alter cell proliferation or invasion ability, and anti-fibrotic property	TGF-β/α-SMA signaling pathway	Qu et al. (2019)
33442414						Iwata et al. (2021)
31254244	Oral squamous cell carcinoma	Increased	Not mentioned	(Controversial) Antagonist to laminin binding, decrease proliferation and invasion; promote cell growth, regulate cell cycle arrest, and inhibit apoptosis	laminin receptor pathway	Hano et al. (2019)
34906149					APR and TNF-α pathway	Song et al. (2021)
33123583	Bladder cancer	Increased	Poor prognosis	Promote cell migration and invasion	ErbB, mTOR, Notch, TGF-β signaling pathway	Zhu et al. (2020)
32282241	Renal cell carcinoma	Increased	Poor prognosis	Serve as an independent risk factor for overall survival, promote cell proliferation, migration, invasion, and inhibit apoptosis	WNT beta catenin signaling pathway	Lin et al. (2020)
26648301	Ovarian cancer (article in Chinese)	Decreased	Not mentioned	Inhibit cell proliferation and migration	IL-8/VEGF pathway	Wang et al. (2015)

MEK, mitogen-activated protein kinase; ERK, extracellular signal-regulated kinase; MAPK, mitogen-activated protein kinases; APR, acute phase response; TGF-β, transforming growth factor-beta; ErbB, epidermal growth factor receptor; IL-8, interleukin-8; VEGF, vascular endothelial growth factor.

## Expression of CTRP6 in humans and mice

**FIGURE 2:**
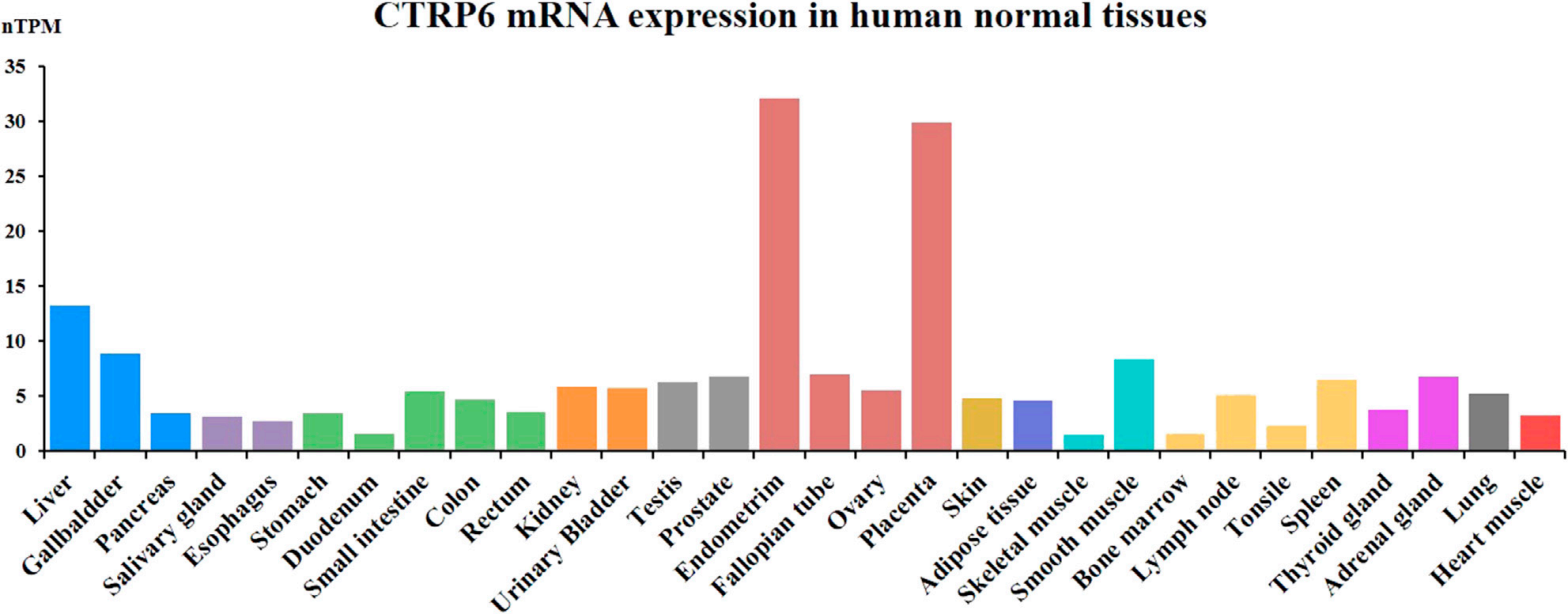
CTRP6 mRNA expression in human normal tissues.

CTRP6 as a secreted protein is widely expressed in various human normal tissues and cell types. CTRP6 protein is mainly expressed in female normal tissue and partially expressed in the gastrointestinal tract (Figure 2). The CTRP6 expression data are from the Human Protein Atlas (HPA) dataset and Genotype-Tissue Expression (GTE) transcriptomics datasets. In female tissues, CTRP6 protein is most highly expressed in the placenta endothelial cells and syncytiotrophoblasts. CTRP6 regulated the viability, migration, and invasion of human chorion trophoblast cells through PPARγ signaling (Zhang and Bai, 2022). In the endometrium, stroma cells but not glandular cells show high expression of CTRP6. In colon and rectum tissue, mucosal lymphoid cells but not glandular or endothelial cells show positive staining of CTRP6. However, most normal tissues do not express CTRP6 protein, and CTRP6 mRNA levels in various tissues are fairly low.

CTRP6 was found in serum and its expression in fat tissues was enhanced in obese, ob/ob and adiponectin null-mice (Liu et al., 2021). However, mRNA levels in the fat of female mice were about five times that of male mice (Liu et al., 2021). Most CTRP family members are predominantly expressed in adipose tissue, but CTRP6 is highly expressed in the placenta tissue. CTRP6 as a secreted glycoprotein is expressed in mammalian cells. Serum level of CTRP6 expression also seems to be increased in female mice, indicating that CTRP6 serum levels vary with the sex and genetic background of mice (Wong et al., 2008). Administration of the peroxisome-proliferator-activated receptor-γ (PPAR-γ) agonist rosiglitazone could significantly reduce the transcript level of CTRP6 in the adipose tissue (Wong et al., 2008). CTRP6 is a gene that can rapidly respond to acute nutritional changes by regulating adipose tissue expansion in mice (Lahav et al., 2021). CTRP6 reduced cerebral ischemia/reperfusion injury (IRI) by decreasing inflammation, oxidative stress, and apoptosis by activating the PI3K/Akt signaling pathway in mice (Li et al., 2020).

## CTRP6 in lung cancer

**FIGURE 3:**
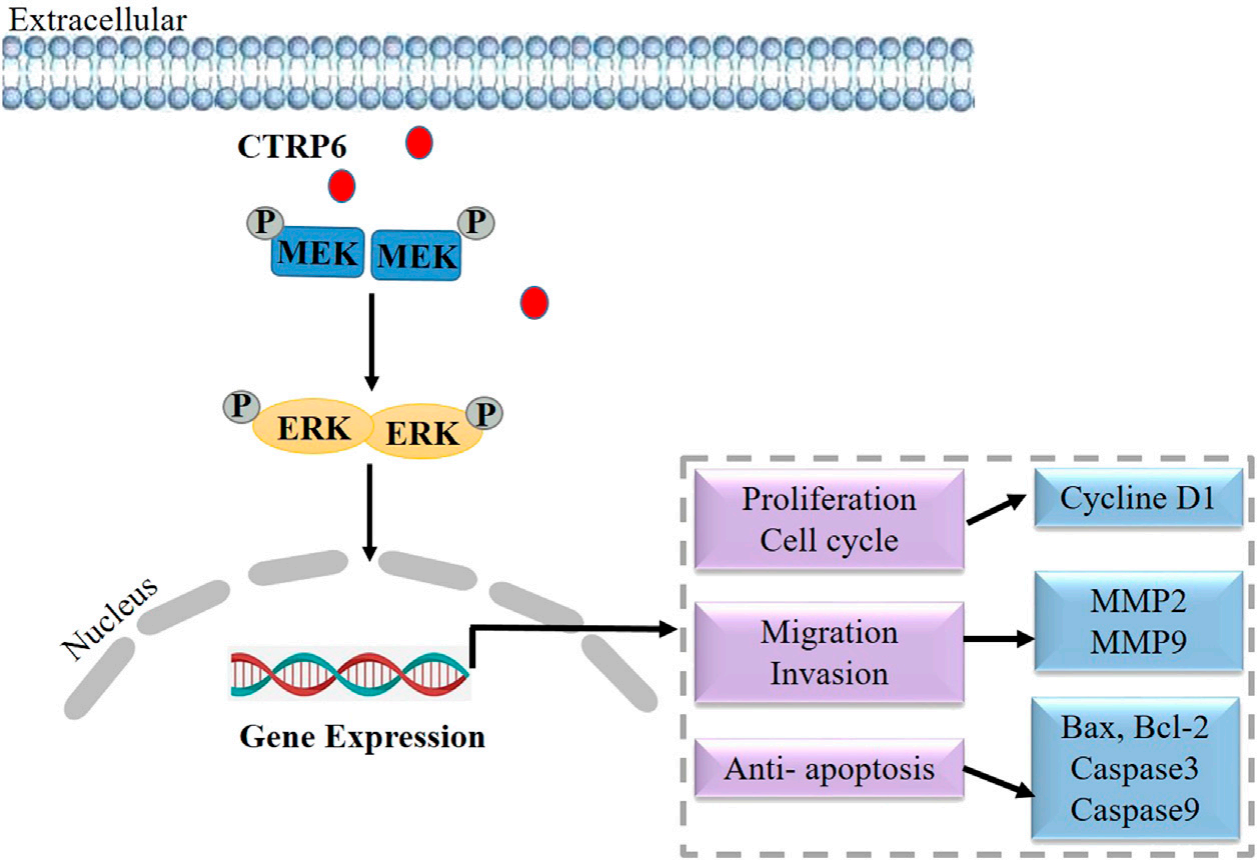
Schematic diagram of CTRP6 mediated the development and progression of lung cancer.

Lung cancer is the most common cause of cancer-related death worldwide, with an estimated 1.6 million deaths each year (Sung et al., 2021). The most common histological subtypes of lung cancer are collectively referred to as non-small cell lung cancer (NSCLC), in which lung adenocarcinoma and lung squamous cell carcinoma are the most common pathologic subtypes (Herbst et al., 2018). Although remarkable progress has been made in the treatment of NSCLC over the past two decades, the overall cure rate and survival rate remain low (Chen et al., 2014). A better understanding of disease biology and tumor progression mechanism can help invent novel treatment strategies earlier. However, the function of CTRP6 in lung adenocarcinoma remains unknown. At present, only two literature studies have reported the biological role of CTRP6 in lung cancer (Han et al., 2019; Zhang and Feng, 2021). Based on the data from TCGA and Oncomine databases, the CTRP6 expression is dramatically up-regulated in human lung adenocarcinoma tissues as compared with that in normal lung samples (Han et al., 2019). CTRP6 expression is positively correlated with tumor T-stage, the number of metastatic lymph nodes, and the distal metastasis status (Han et al., 2019). In survival analysis, CTRP6 is significantly associated with overall survival (OS) in lung adenocarcinoma patients. The statistical data show that CTRP6 high-expression group is related to an unfavorable prognosis as compared with CTRP6 low-expression group (Han et al., 2019). It is suggested that CTRP6 can be used as an independent predictor of lung adenocarcinoma prognosis. CTRP6 expression was found to be increased in two different lung cancer cell lines (Han et al., 2019). CTRP6 expression at mRNA and protein levels are up-regulated in H1299 and A549 lung cancer cells as compared with BEAS-2B normal control. Knockdown of CTRP6 inhibited the capabilities of cell proliferation, migration, and invasion in lung adenocarcinoma cells. Additionally, knockdown of CTRP6 showed a positive regulatory effect on MEK and ERK phosphorylation (Han et al., 2019) but did not affect MEK and ERK protein expression. MEK and ERK are two key cascades in the MAPK signaling pathway in NSCLC (Han et al., 2021). Activating the RAS/RAF/MEK/ERK signaling pathway leads to the proliferation, and regulated cell cycle of cancer cells (Liang et al., 2021). Inactivating MAPK signaling pathway showed a negative regulatory effect on the progression and metastasis of lung cancer (Xu et al., 2018). These findings suggest that CTRP6 may promote the progression of lung cancer *via* regulating the MAPK signaling pathway (Figure 3).

## CTRP6 in hepatocellular carcinoma

Hepatocellular carcinoma (HCC), the most common type of liver cancer, is leading to a growing global problem with one of the worse prognoses (Llovet et al., 2021). Viral infections and the increasing incidence of obesity and fatty liver disease also contribute to liver carcinogenesis (Marengo et al., 2016). Despite the development of new therapeutic drugs over the past few years, OS does not improve further (Couri and Pillai, 2019). Like other cancers, multiple signaling pathways have been implicated in HCC, which provide more accurate and effective therapeutic methods for tumor treatment (Zhao et al., 2020).

Previous studies have reported an increased expression of CTRP6 in human HCC tissue (Wan et al., 2019). Takeuchi et al. (2011) reported their immunohistochemical (IHC) study on 30 HCC tissue specimens and found that 21 of these specimens had positive staining, but no increased expression of CTRP6 was detected in the peritumoral liver tissue (Takeuchi et al., 2011). Positive CTRP6 IHC staining was not only found in the cytoplasm of tumor cells but present in sinusoidal lining cells. CTRP6 mRNA and protein expression levels of human HCC were also detected by RT-PCR and Western blot, demonstrating that CTRP6 expression was significantly up-regulated in the HCC tissue as compared with that in paratumoral liver tissue (Wan et al., 2019).

An *in vivo* study using the experimental model of subcutaneous injection of HepG2 cells in BALB/c nude mice (Takeuchi et al., 2011) showed that enhancing the expression of CTRP6 can accelerate tumor neovascularization in xenografts. Representative histological features of typical xenografts showed that the central area of hypovascular tumor necrosis in non-CTRP6 expressing HepG2 cells was larger than that in CTRP6-expression HepG2 cells. Meanwhile, the CTRP6-expression HepG2 cells showed a rare necrotic area with abundant tumor-penetrating vessels in the formed solid tumors. But there was no significant difference in tumor volumes between these two groups. In conclusion, xenograft assay showed that CTRP6 can help tumor growth by promoting angiogenesis in the tumor, thereby decreasing the hypovascular central necrosis area in transplanted HepG2 cells.

**FIGURE 4:**
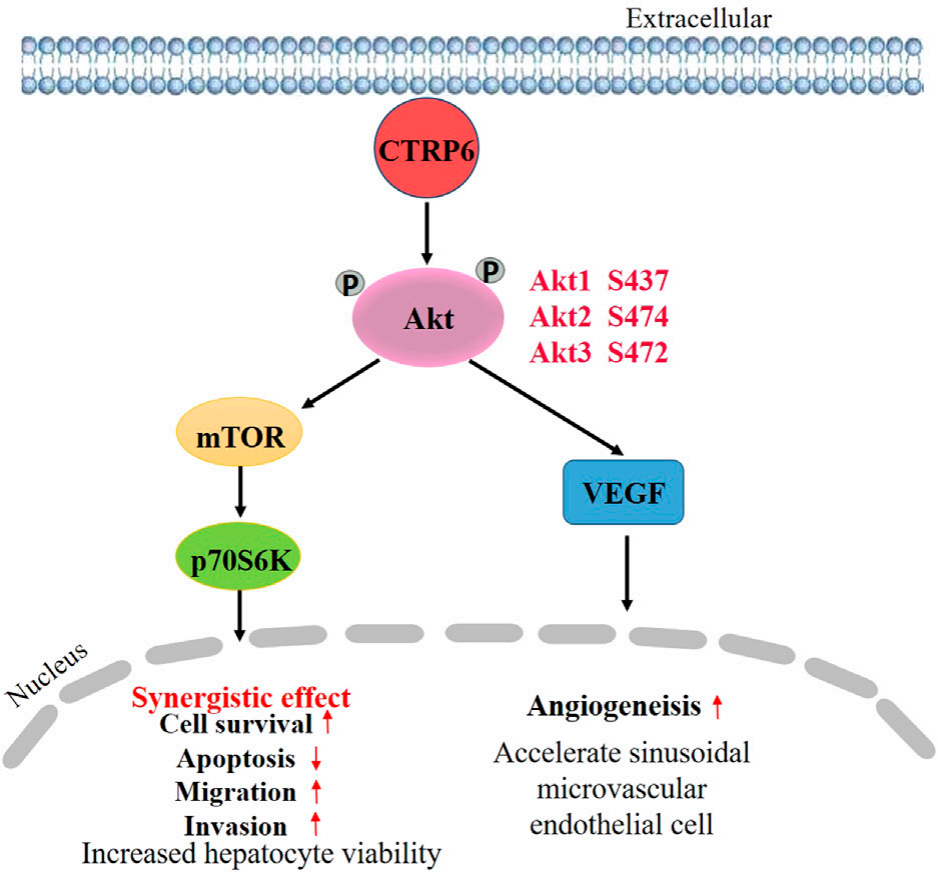
Schematic diagram of CTRP6 mediated promotion of hepatocellular carcinoma growth.

CTRP6 was highly expressed in the human HCC cell line Hep3B in comparison with normal human liver cell line L02 (Wan et al., 2019). Silencing CTRP6 by siRNA transfection could inhibit cell viability, and promote the apoptosis of Hep3B cells (Wan et al., 2019). Caspase-3/CPP32 fluorometric assay showed that knocking down CTRP6 enhanced the activity of caspase-3. In addition, depletion of CTRP6 reduced the ability of cell migration and invasion (Wan et al., 2019). To better clarify the potential mechanism of CTRP6 in HCC cell biological behaviors, the role of CTRP6 in the Akt signaling pathway has attracted more attention. It is well known that Akt/mTOR is frequently mutated in HCC (Rebouissou and Nault, 2020). Activation of Akt can help tumor growth and metastasis in HCC (Dimri and Satyanarayana, 2020). Akt phosphorylation is known to promote cell growth and survival in a variety of solid tumors including HCC by activating the PI3K/Akt/mTOR pathway. Activation of the PI3K/Akt-pathway is one of the key mechanisms in HCC (Wu et al., 2020). PI3K/Akt pathway also acts an important role in growth control and drug resistance in cancer cells (Garcia-Lezana et al., 2021). An *in vitro* study (Wan et al., 2019) showed that CTRP6 increased the phosphorylation of Akt in cultured human liver sinusoidal microvascular endothelial cells. Meanwhile, silencing CTRP6 expression could decrease the phosphorylation level of Akt. The C1 domain at the C-terminal side of CTRP6 seems to be critical for CTRP6-mediated Akt activation (Wan et al., 2019). CTRP6 may promote tumor angiogenesis in HCC by activating the Akt pathway in vascular endothelial cells. Inhibition of Akt phosphorylation decreased angiogenesis of HCC (Xie et al., 2021). Additionally, hepatic angiogenesis can be regulated by VEGF/Akt/eNOS signaling pathway (Zheng et al., 2021). Moreover, pretreatment of Hep3B cells with insulin-like growth factor 1 (IGF-1), an activator of Akt, could restore the changes in cell biological behavior by CTRP6-siRNA transfection. Activating Akt signaling pathway reverses the alterations after CTRP6 inhibition, including cell survival, apoptosis, migration, and invasion, indicating that CTRP6 is involved in HCC progression *via* the mediation of Akt signaling pathway (Figure 4).

## CTRP6 in gastric cancer

Gastric cancer (GC) is the fifth most prevalent cancer and the third leading cause of cancer mortality worldwide (Sung et al., 2021). There are many histological classifications of GC. According to the Lauren classification system, GC is classified into two types: intestinal and diffuse. Although GC can be surgically excised or inhibited by chemoradiotherapy, the prognosis remains poor in many cases because of the advanced stage at the time of diagnosis (Smyth et al., 2020). Previous studies (Qu et al., 2019; Iwata et al., 2021) have reported that the expression of CTRP6 in GC is increased. Comparison of CTRP6 mRNA alteration by GeneChip assay demonstrated that the expression of CTRP6 mRNA in human GC was significantly up-regulated by 1.59 fold as compared with that in the peritumoral normal gastric tissue. IHC detection of CTRP6 protein in primary GC samples showed a serum immunoreactive expression pattern and a diffuse granular distribution (Qu et al., 2019). The expression rate of CTRP6 was about 80.7% (42/52) and 46.9% (23/49) in two studies respectively (Qu et al., 2019; Iwata et al., 2021). Interestingly, although CTRP6 was found in advanced gastric cancer, invasion front of gastric cancer cells showed a loss of CTRP6 expression in diffuse gastric adenocarcinoma specimens (Iwata et al., 2021). CTRP6 is highly expressed in GC tissue, regardless of patient age and sex, tumor location, degree of differentiation, depth of invasion, lymph node metastasis, lymph vascular involvement, and nerve infiltration. CTRP6 expression was found to be significantly correlated with a favorable recurrence-free period (RFP) in patients with distal diffuse gastric adenocarcinoma (Iwata et al., 2021).

**FIGURE 5:**
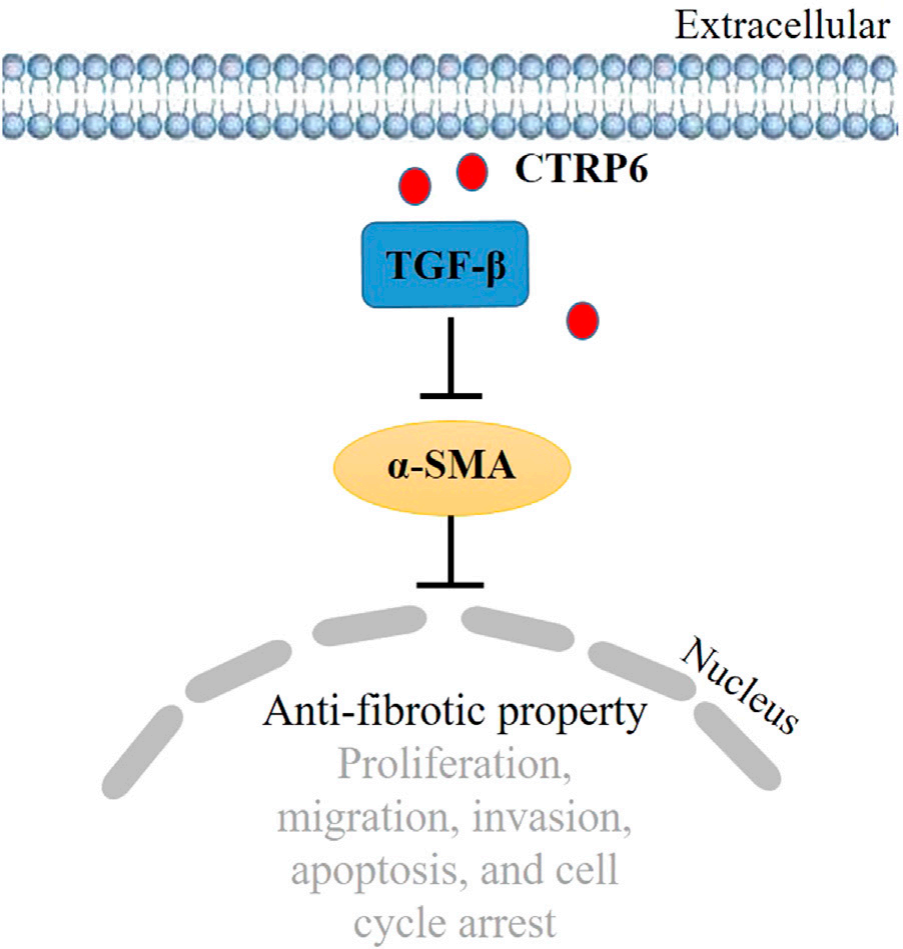
Schematic diagram of the role of CTRP6 in gastric cancer.

CTRP6 expression was obtained in many human GC cell lines, including AGS, SSGC-7901, BGC-823, MGC-803, KATO III, MKN7, MKN74, NUGC4, and GPM-2. But the role of CTRP6 in GC remains controversial. Silencing CTRP6 expression decreased cell proliferation and the ability of cell migration and invasion (Qu et al., 2019). It was also reported that CTRP6 knockdown could induce cell cycle arrest and promote apoptosis of AGS cells (Qu et al., 2019). However, CTRP6 was found to play a dual role in GC study. A previous study (Iwata et al., 2021) showed that treatment with recombinant CTRP6 did not change the viability of GC cells, and neither did the primary cultured normal gastric epithelial cells. In addition, it was reported that treatment of recombinant CTRP6 could significantly reduce the TGF-β induced alpha-smooth muscle actin (α-SMA) expression in fibroblasts (Figure 5). TGF-β induced α-SMA promoted tumor cell invasion and metastasis (Fuyuhiro et al., 2011). A subpopulation of cancer-associated fibroblasts (CAFs) is known to play a major role in the tumor microenvironment (TME). It was found that CAFs with α-SMA protein expression profile enhanced the invasiveness of cancer cells (Naito et al., 2019). Additionally, high expression of α-SMA was associated with poor prognosis in gastric adenocarcinoma patients (Zhan et al., 2019). Inverse expression of CTRP6 and α-SMA was also obtained by IHC assay in human GC specimens, indicating that CTRP6 could attenuate the invasiveness of α-SMA positive fibroblasts in cancer stroma. The results of previous studies on the function of CTRP6 in GC are controversial, and therefore it is necessary to perform further investigations to elucidate the role of CTRP6 in GC.

## CTRP6 in oral squamous cell carcinoma

Oral squamous cell carcinoma (OSCC) is one of the most common head and neck malignant tumors (Warnakulasuriya, 2009). The 5-year OS of OSCC has remains 40%–60% without significant improvement over the past four decades (Zanoni et al., 2019). So far, only two studies have reported the expression of CTRP6 in human OSCC (Hano et al., 2019; Song et al., 2021), pointing out that CTRP6 played a bidirectional role in the carcinogenesis of OSCC. The result from The Cancer Genome Atlas (TCGA) shows that CTRP6 tended to be more highly expressed in OSCC of 40 paired cancer and normal tissues (Song et al., 2021). This finding was also verified by qRT-PCR and western blot analysis, demonstrating that the expression level of CTRP6 in cancer tissue was significantly higher than that in adjacent normal tissues (Song et al., 2021). In addition, IHC staining also confirmed the consistent results that CTRP6 expression was elevated in OSCC cancer cells. High CTRP6 expression was also found in Cal-27 and SCC-9 human OSCC cell lines, but not HioEC normal oral cell lines. Knockdown of CTRP6 can inhibit cell proliferation and enhance cell apoptosis in OSCC cell lines. Two different *in vivo* studies proved that CTRP6 could slow down the growth of OSCC cells in the xenograft model. One study established the model by injecting OSCC cells transfected with CTRP6-shRNA or Ctrl-shRNA lentivirus into nude mice (Song et al., 2021), and another study established the xenograft model by intraperitoneal injection of inoculating into the mice and treated them with or without recombinant CTRP6 protein (Hano et al., 2019). The result showed that the tumor volume in the CTRP6 group was significantly smaller than that in the control group, indicating that CTRP6 could suppress OSCC cell proliferation *in vivo* (Hano et al., 2019).

**FIGURE 6:**
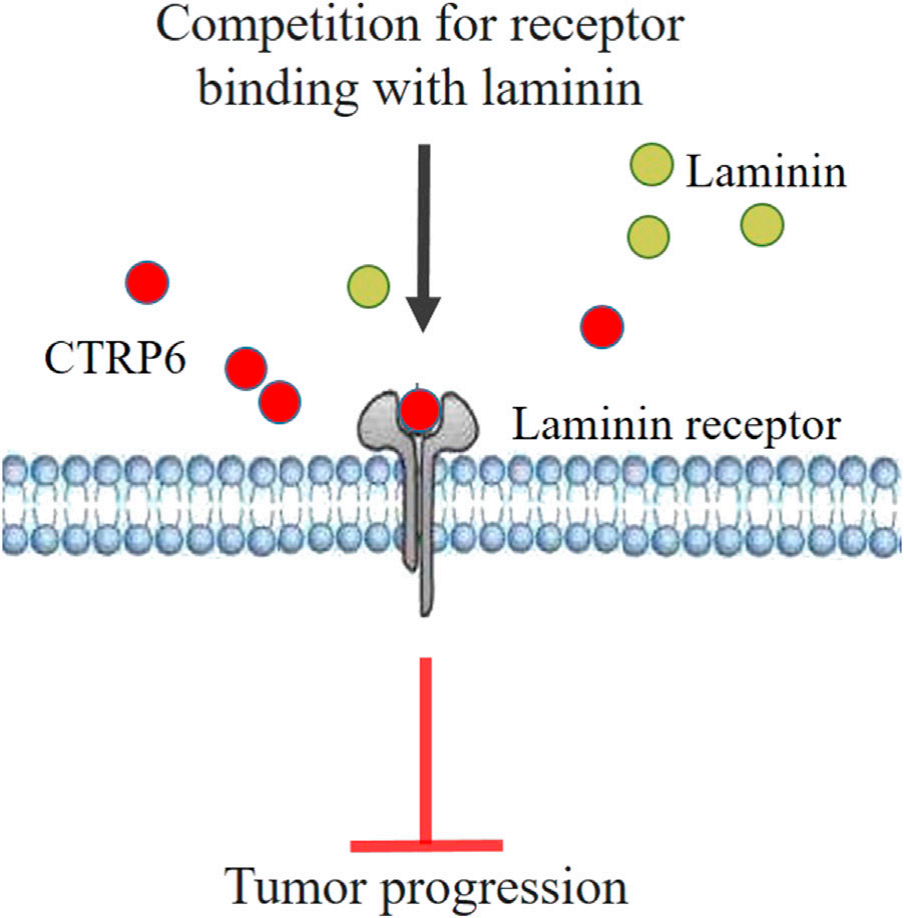
Hypothetical model of CTRP6 mediated tumor suppression mechanism in oral squamous cell carcinoma.

An *in vitro* study (Hano et al., 2019) demonstrated that administration of recombinant CTRP6 could significantly inhibit cell proliferation and invasion ability in cultured OSCC cells. Microarray analysis reveals that silencing CTRP6 can significantly activate the acute phase response signaling pathway, and the upstream molecules, such as ID1, BBC3, and DDIT3, were closely related to CTRP6 in the network (Song et al., 2021). ID1 showed increased expression in OSCC (Chen et al., 2020). In addition, ID1 expression was significantly associated with tumor recurrence, angiogenesis, lymph node metastasis, and poor clinical outcome (Dong et al., 2010). Lu et al. (2020) reported that ID1 activation promoted the stemness of OSCC cells. The mechanism of CTRP6 inhibiting tumor growth assumed that CTRP6 inhibited the growth of OCSS by binding to a cell surface membrane receptor (Hano et al., 2019). Subsequent screening analysis revealed that CTRP6 is bound to the product of human ribosomal protein SA (RPSA), which is also known as the laminin receptor (37LRP/67LR) gene. The CTRP6 binding protein was screened in the expression library and then analyzed by co-immunoprecipitation. The results showed that CTRP6 is combined with the laminin receptor precursor in the surface membrane of cultured human OSCC SAS cells (Hano et al., 2019). It is suggested that CTRP6 acts as an antagonist to laminin binding in OCSS. The binding of laminin and its receptor is considered to attenuate the migration and invasion of cancer cells (Hano et al., 2019). Increased laminin-α3 mutations are associated with carcinogenesis and invasiveness of OSCC (Sequeira et al., 2020). High laminin receptor expression is considered a useful prognostic factor associated with poor prognosis and postoperative recurrence of SCC (Matsuo et al., 2022). CTRP6 protein significantly suppressed the migration and matrigel invasion activity of ASA cells (Hano et al., 2019). The mechanism of CTRP6 inhibiting cell migration and invasion may be that CTRP6 disturbs the binding of laminin to the laminin receptor (Figure 6). CTRP6 may therefore be considered a novel potential approach for OSCC treatment.

## CTRP6 in bladder cancer

Bladder cancer (BC) is one of the most prevalent malignancies in the urinary system and is classified as the fourth major type of cancer in men worldwide (Lenis et al., 2020). The risk factors of BC are complex and the mechanisms underlying its development and progression remain unclear (Grayson, 2017). CTRP6 is recently discovered as a novel molecule for predicting the prognosis of BC (Zhu et al., 2020). According to TCGA and GEO databases, the CTRP6 mRNA levels in the BC tissue increased significantly as compared with that in normal bladder mucosa (Zhu et al., 2020). CTRP6 protein expression in BC tissue was obtained by the HPA database. The IHC expression pattern of CTPR6 showed cytoplasmic stains in tumor cells. In addition, the expression of CTRP6 is positively correlated with an advanced AJCC stage, a higher histologic grade, and a worse prognosis in BC. In addition, the high CTRP6 expression group presented poor OS and progression-free survival (PFS) in both TCGA and GEO databases (Zhu et al., 2020). Survival analysis demonstrated that patients in the low CTRP6 expression group had a significantly favorable OS as compared with that in the high CTRP6 expression group. Gene set enrichment analysis (GSEA) of CTRP6 expression was detected to evaluate the potential correlations between CTRP6 and relevant pathways, and the result showed that cytokine-cytokine receptor interaction, ErbB signaling pathway, and ECM receptor interaction were enriched in high CTRP6 expression group (Zhu et al., 2020). In addition, CTRP6 low-expression group was assumed enriched in some cancer related pathways, including Notch, TFG-β, mTOR, and ubiquitin-mediated proteolysis. The expression of ECM-related genes was significantly higher in the tumor-associated urothelium than that in the urothelium (Wullweber et al., 2021). ErbB is found overexpressed in a subgroup of BC, and targeting the ErbB/HER receptors in patients is considered to be a new strategy treatment (Chen et al., 2021). Compared with normal bladder cell line (SV-HUCL), increased expression of CTRP6 was also detected in several BC cell lines, including T24, UMUC3, BIU87, and 5637 (Zhu et al., 2020). It was found that depletion of CTRP6 expression by siRNA transfection could reduce the migration and invasion abilities of BC cells. However, the underlying function and molecular mechanism of CTRP6 in the biological behaviors of bladder tumor cells need to be further investigated.

## CTRP6 in renal cancer

Clear cell renal cell carcinoma (ccRCC) is one of the major subtypes of renal cell cancer, with a prevalence of 75% in all primary kidney cancers (Hsieh et al., 2017). The ccRCC is a highly aggressive disease, and up to one-third of ccRCC patients were diagnosed in the advanced tumor stage without typical symptoms (Motzer et al., 2022). TCGA and GEO databases showed that CTRP6 expression was up-regulated in the ccRCC samples compared with the adjacent normal tissue (Lin et al., 2020). Besides, high levels of CTRP6 mRNA and protein expression were found to be positively correlated with cancer stages. The expression level of CTPR6 tends to be high in patients with advanced cancer stage and its Overexpression is significantly correlated with poor OS in ccRCC patients (Lin et al., 2020). Univariate and multivariate Cox analyses showed that CTPR6 was an independent risk factor for OS. KEGG and GSEA analyses showed that co-expressed genes and signaling pathways in the CTRP6 high expression group are mainly enriched in cell cycle, DNA replication, epithelial-mesenchymal transition (EMT), angiogenesis, and WNT/β-catenin signaling pathways (Lin et al., 2020). In addition, activation of the WNT/β-catenin signaling pathway could favorably trigger the EMT in CRCC (Gorka et al., 2021). All these networks are closely related to tumor cell proliferation, migration, invasion, and metastasis, but more experimental results are needed to verify the functional role of CTRP6 in renal malignancies. In conclusion, all the above results indicate that CTRP6 can be used as a potentially useful biomarker to predict the survival of ccRCC patients. However, the results of the CTRP6 research are limited, and most results were obtained by bioinformatics analysis. Further *in vivo* and *in vitro* experimental studies are needed to explore the biological function of CTRP6 in ccRCC.

## CTRP6 in other tumors

In addition, the role of CTRP6 in regulating tumor progression has also been reported in ovarian cancer (Wang et al., 2015) and colon cancer (Jingxian Gou et al., 2019). Both articles are in Chinese. The level of CTRP6 in the serum of patients with ovarian cancer showed a declining trend. Contrary to the previously described function, CTRP6 restrained the proliferation and migration of ovarian cancer cells (Wang et al., 2015). The inhibitory effect of CTRP6 can be attenuated by treatment with CTRP6 siRNA or anti-CTRP6 antibody. CTRP6 inhibited the viability and migration of ovarian cancer cells by blocking the expression of IL-8 and vascular endothelial growth factor (VEGF) pathways. CTRP6 is overexpressed in colon cancer tissue (Jingxian Gou et al., 2019). But no significant relationship was observed between CTRP6 expression and patients’ gender, age, tumor size, tumor differentiation, and depth of tumor invasion. Still, there is no comprehensive study on the mechanism of CTRP6 in colon cancer and other tumors.

## Conclusion

**FIGURE 7:**
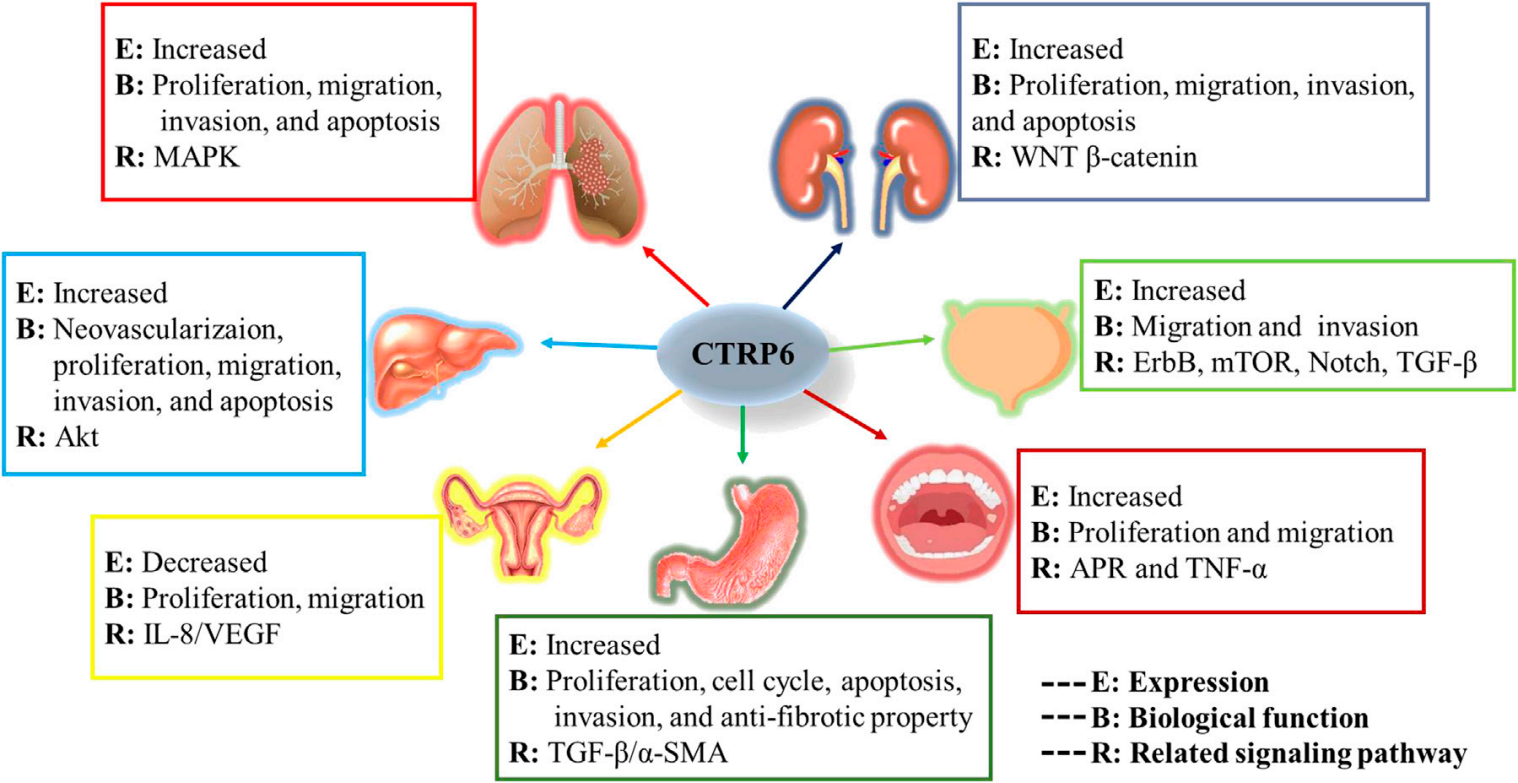
Graphic summary of CTRP6 in various tumor types.

In the current review, we investigate the role of CTRP6 in various malignant tumors (Figure 7). We focused on its expression, biological function, and main related signaling pathways in different malignancies. The study of biochemical characterization of CTRP6 and its heteromeric protein complexes will help us further understand the complex pathophysiological functions of CTRP6.

Studies have identified CTRP6 as a therapeutic target for a variety of cancers, which brings more attention and hope for developing potential therapeutic strategies. CTRP6 seems to play a dual role in different tumors. However, the list of CTRP6 related malignancies continues to expand, and research on CTRP6-related tumor diseases remains insufficient. More research is required to gain a better understanding of the biological function of CTRP6 in various tumors.

## References

[B1] Akiyama H. Furukawa S. Wakisaka S. Maeda T. (2009). Elevated expression of CTRP3/cartducin contributes to promotion of osteosarcoma cell proliferation. Oncol. Rep. 21 (6), 1477–1481. 10.3892/or_00000377 19424626

[B2] Avgerinos K. I. Spyrou N. Mantzoros C. S. Dalamaga M. (2019). Obesity and cancer risk: Emerging biological mechanisms and perspectives. Metabolism. 92, 121–135. 10.1016/j.metabol.2018.11.001 30445141

[B3] Chen D. Ye Y. Guo S. Yao K. (2021). Progress in the research and targeted therapy of ErbB/HER receptors in urothelial bladder cancer. Front. Mol. Biosci. 8, 800945. 10.3389/fmolb.2021.800945 35004854 PMC8735837

[B4] Chen J. Zhang F. Wang D. Yang Z. Liu S. Dong Z. (2020). Prognostic ability of DNA-binding protein inhibitor ID-1 expression in patients with oral squamous cell carcinoma. Oncol. Lett. 19 (6), 3917–3922. 10.3892/ol.2020.11506 32382338 PMC7202274

[B5] Chen Z. Fillmore C. M. Hammerman P. S. Kim C. F. Wong K.-K. (2014). Non-small-cell lung cancers: A heterogeneous set of diseases. Nat. Rev. Cancer 14 (8), 535–546. 10.1038/nrc3775 25056707 PMC5712844

[B6] Couri T. Pillai A. (2019). Goals and targets for personalized therapy for HCC. Hepatol. Int. 13 (2), 125–137. 10.1007/s12072-018-9919-1 30600478

[B7] De Pergola G. Silvestris F. (2013). Obesity as a major risk factor for cancer. J. Obes. 2013, 291546. 10.1155/2013/291546 24073332 PMC3773450

[B8] Di Zazzo E. Polito R. Bartollino S. Nigro E. Porcile C. Bianco A. (2019). Adiponectin as link factor between adipose tissue and cancer. Int. J. Mol. Sci. 20 (4), E839. 10.3390/ijms20040839 30781341 PMC6412253

[B9] Dimri M. Satyanarayana A. (2020). Molecular signaling pathways and therapeutic targets in hepatocellular carcinoma. Cancers 12 (2), E491. 10.3390/cancers12020491 32093152 PMC7072513

[B10] Dong X. Hu H. Fang Z. Cui J. Liu F. (2018). CTRP6 inhibits PDGF-BB-induced vascular smooth muscle cell proliferation and migration. Biomed. Pharmacother. = Biomedecine Pharmacother. 103, 844–850. 10.1016/j.biopha.2018.04.112 29710500

[B11] Dong Z. Liu S. Zhou C. Sumida T. Hamakawa H. Chen Z. (2010). Overexpression of Id-1 is associated with tumor angiogenesis and poor clinical outcome in oral squamous cell carcinoma. Oral Oncol. 46 (3), 154–157. 10.1016/j.oraloncology.2009.11.005 20056476

[B12] Fuyuhiro Y. Yashiro M. Noda S. Kashiwagi S. Matsuoka J. Doi Y. (2011). Upregulation of cancer-associated myofibroblasts by TGF-β from scirrhous gastric carcinoma cells. Br. J. Cancer 105 (7), 996–1001. 10.1038/bjc.2011.330 21863023 PMC3185946

[B13] Garcia-Lezana T. Lopez-Canovas J. L. Villanueva A. (2021). Signaling pathways in hepatocellular carcinoma. Adv. Cancer Res. 149, 63–101. 10.1016/bs.acr.2020.10.002 33579428

[B14] Glogowska A. Thanasupawat T. Beiko J. Pitz M. Hombach-Klonisch S. Klonisch T. (2022). Novel CTRP8-RXFP1-JAK3-STAT3 axis promotes Cdc42-dependent actin remodeling for enhanced filopodia formation and motility in human glioblastoma cells. Mol. Oncol. 16 (2), 368–387. 10.1002/1878-0261.12981 33960104 PMC8763656

[B15] Gorka J. Marona P. Kwapisz O. Waligórska A. Pospiech E. Dobrucki J. W. (2021). MCPIP1 inhibits Wnt/β-catenin signaling pathway activity and modulates epithelial-mesenchymal transition during clear cell renal cell carcinoma progression by targeting miRNAs. Oncogene 40 (50), 6720–6735. 10.1038/s41388-021-02062-3 34657130 PMC8677621

[B16] Grayson M. (2017). Bladder cancer. Nature 551 (7679), S33. 10.1038/551S33a 29117156

[B17] Han J. Liu Y. Yang S. Wu X. Li H. Wang Q. (2021). MEK inhibitors for the treatment of non-small cell lung cancer. J. Hematol. Oncol. 14 (1), 1. 10.1186/s13045-020-01025-7 33402199 PMC7786519

[B18] Han M. Wang B. Zhu M. Zhang Y. (2019). C1QTNF6 as a novel biomarker regulates cellular behaviors in A549 cells and exacerbates the outcome of lung adenocarcinoma patients. Vitro Cell. Dev. Biol. Anim. 55 (8), 614–621. 10.1007/s11626-019-00377-w 31292940

[B19] Hano K. Hatano K. Saigo C. Kito Y. Shibata T. Takeuchi T. (2019). An adiponectin paralog protein, CTRP6 decreased the proliferation and invasion activity of oral squamous cell carcinoma cells: Possible interaction with laminin receptor pathway. Mol. Biol. Rep. 46 (5), 4967–4973. 10.1007/s11033-019-04947-9 31254244

[B20] Herbst R. S. Morgensztern D. Boshoff C. (2018). The biology and management of non-small cell lung cancer. Nature 553 (7689), 446–454. 10.1038/nature25183 29364287

[B21] Hopkins B. D. Goncalves M. D. Cantley L. C. (2016). Obesity and cancer mechanisms: Cancer metabolism. J. Clin. Oncol. 34 (35), 4277–4283. 10.1200/JCO.2016.67.9712 27903152 PMC5562429

[B22] Hsieh J. J. Purdue M. P. Signoretti S. Swanton C. Albiges L. Schmidinger M. (2017). Renal cell carcinoma. Nat. Rev. Dis. Prim. 3, 17009. 10.1038/nrdp.2017.9 28276433 PMC5936048

[B23] Iwata Y. Yasufuku I. Saigo C. Kito Y. Takeuchi T. Yoshida K. (2021). Anti-fibrotic properties of an adiponectin paralog protein, C1q/TNF-related protein 6 (CTRP6), in diffuse gastric adenocarcinoma. J. Cancer 12 (4), 1161–1168. 10.7150/jca.46765 33442414 PMC7797637

[B24] Jingxian Gou X. J. Qu H. Cui Y. (2019). Complement C1q/tumor necrosis factor-related protein 6 expression in colon cancer. Chin. J. Gastroenterol. Hepatol. 28, 5404.

[B25] Jung H. N. Jung C. H. (2021). The role of anti-inflammatory adipokines in cardiometabolic disorders: Moving beyond adiponectin. Int. J. Mol. Sci. 22 (24), 13529. 10.3390/ijms222413529 34948320 PMC8707770

[B26] Klonisch T. Glogowska A. Thanasupawat T. Burg M. Krcek J. Pitz M. (2017). Structural commonality of C1q TNF-related proteins and their potential to activate relaxin/insulin-like family peptide receptor 1 signalling pathways in cancer cells. Br. J. Pharmacol. 174 (10), 1025–1033. 10.1111/bph.13559 27443788 PMC5406291

[B27] Kong M. Gao Y. Guo X. Xie Y. Yu Y. (2021). Role of the CTRP family in tumor development and progression. Oncol. Lett. 22 (4), 723. 10.3892/ol.2021.12984 34429763 PMC8371956

[B28] Lahav R. Haim Y. Bhandarkar N. S. Levin L. Chalifa-Caspi V. Sarver D. (2021). CTRP6 rapidly responds to acute nutritional changes, regulating adipose tissue expansion and inflammation in mice. Am. J. Physiol. Endocrinol. Metab. 321 (5), E702–E713. 10.1152/ajpendo.00299.2021 34632797 PMC8799396

[B29] Lei H. Wu D. Wang J.-Y. Li L. Zhang C.-L. Feng H. (2015). C1q/tumor necrosis factor-related protein-6 attenuates post-infarct cardiac fibrosis by targeting RhoA/MRTF-A pathway and inhibiting myofibroblast differentiation. Basic Res. Cardiol. 110 (4), 35. 10.1007/s00395-015-0492-7 25962701

[B30] Lei X. Seldin M. M. Little H. C. Choy N. Klonisch T. Wong G. W. (2017). C1q/TNF-related protein 6 (CTRP6) links obesity to adipose tissue inflammation and insulin resistance. J. Biol. Chem. 292 (36), 14836–14850. 10.1074/jbc.M116.766808 28726640 PMC5592665

[B31] Lengyel E. Makowski L. DiGiovanni J. Kolonin M. G. (2018). Cancer as a matter of fat: The crosstalk between adipose tissue and tumors. Trends Cancer 4 (5), 374–384. 10.1016/j.trecan.2018.03.004 29709261 PMC5932630

[B32] Lenis A. T. Lec P. M. Chamie K. Mshs M. D. (2020). Bladder cancer: A review. JAMA 324 (19), 1980–1991. 10.1001/jama.2020.17598 33201207

[B33] Li Q. Wang L. Tan W. Peng Z. Luo Y. Zhang Y. (2011). Identification of C1qTNF-related protein 4 as a potential cytokine that stimulates the STAT3 and NF-κB pathways and promotes cell survival in human cancer cells. Cancer Lett. 308 (2), 203–214. 10.1016/j.canlet.2011.05.005 21658842

[B34] Li Y. Sun J. Gu L. Gao X. (2020). Protective effect of CTRP6 on cerebral ischemia/reperfusion injury by attenuating inflammation, oxidative stress and apoptosis in PC12 cells. Mol. Med. Rep. 22 (1), 344–352. 10.3892/mmr.2020.11108 32377750 PMC7248524

[B35] Li Y. Wright G. L. Peterson J. M. (2017). C1q/TNF-Related protein 3 (CTRP3) function and regulation. Compr. Physiol. 7 (3), 863–878. 10.1002/cphy.c160044 28640446 PMC5756469

[B36] Liang Y. Zhang T. Jing S. Zuo P. Li T. Wang Y. (2021). 20(S)-Ginsenoside Rg3 inhibits lung cancer cell proliferation by targeting EGFR-mediated ras/raf/MEK/ERK pathway. Am. J. Chin. Med. 49 (3), 753–765. 10.1142/S0192415X2150035X 33641655

[B37] Liao X. Liu S. Tang X. Yang D. Liu H. Gao L. (2021). Circulating CTRP6 levels are increased in overweight or obese Chinese individuals and associated with insulin resistance parameters: A pilot study. Exp. Clin. Endocrinol. Diabetes 129 (7), 535–541. 10.1055/a-0929-6072 31412378

[B38] Lin W. Chen X. Chen T. Liu J. Ye Y. Chen L. (2020). C1QTNF6 as a novel diagnostic and prognostic biomarker for clear cell renal cell carcinoma. DNA Cell Biol. 39 (6), 1000–1011. 10.1089/dna.2019.5299 32282241

[B39] Liu J. Yan X. Wang Z. Zhang N. Lin A. Li Z. (2021). Adipocyte factor CTRP6 inhibits homocysteine-induced proliferation, migration, and dedifferentiation of vascular smooth muscle cells through PPARγ/NLRP3. Biochem. Cell Biol. = Biochimie Biol. Cell. 99 (5), 596–605. 10.1139/bcb-2020-0670 34469206

[B40] Llovet J. M. Kelley R. K. Villanueva A. Singal A. G. Pikarsky E. Roayaie S. (2021). Hepatocellular carcinoma. Nat. Rev. Dis. Prim. 7 (1), 16018. 10.1038/nrdp.2016.18 PMC13537433 33479224

[B41] Lu L. Wang P. Zou Y. Zha Z. Huang H. Guan M. (2020). IL-1β promotes stemness of tumor cells by activating smad/ID1 signaling pathway. Int. J. Med. Sci. 17 (9), 1257–1268. 10.7150/ijms.44285 32547321 PMC7294920

[B42] Marengo A. Rosso C. Bugianesi E. (2016). Liver cancer: Connections with obesity, fatty liver, and cirrhosis. Annu. Rev. Med. 67, 103–117. 10.1146/annurev-med-090514-013832 26473416

[B43] Matsuo K. Akiba J. Ogasawara S. Kondo R. Naito Y. Kusano H. (2022). Expression and significance of laminin receptor in squamous cell carcinoma of the tongue. J. Oral Pathol. Med. 51 (3), 263–271. 10.1111/jop.13247 34581463

[B44] Motzer R. J. Jonasch E. Agarwal N. Alva A. Baine M. Beckermann K. (2022). Kidney cancer, version 3.2022, NCCN clinical practice guidelines in oncology. J. Natl. Compr. Canc. Netw. 20 (1), 71–90. 10.6004/jnccn.2022.0001 34991070 PMC10191161

[B45] Murayama M. A. Kakuta S. Inoue A. Umeda N. Yonezawa T. Maruhashi T. (2015). CTRP6 is an endogenous complement regulator that can effectively treat induced arthritis. Nat. Commun. 6, 8483. 10.1038/ncomms9483 26404464 PMC4598845

[B46] Naito Y. Yamamoto Y. Sakamoto N. Shimomura I. Kogure A. Kumazaki M. (2019). Cancer extracellular vesicles contribute to stromal heterogeneity by inducing chemokines in cancer-associated fibroblasts. Oncogene 38 (28), 5566–5579. 10.1038/s41388-019-0832-4 31147602 PMC6755971

[B47] Omeka W. K. M. Liyanage D. S. Priyathilaka T. T. Kwon H. Lee S. Lee J. (2019). Characterization of four C1q/TNF-related proteins (CTRPs) from red-lip mullet (Liza haematocheila) and their transcriptional modulation in response to bacterial and pathogen-associated molecular pattern stimuli. Fish. Shellfish Immunol. 84, 158–168. 10.1016/j.fsi.2018.09.078 30287348

[B48] Peterson J. M. Wei Z. Wong G. W. (2009). CTRP8 and CTRP9B are novel proteins that hetero-oligomerize with C1q/TNF family members. Biochem. Biophys. Res. Commun. 388 (2), 360–365. 10.1016/j.bbrc.2009.08.014 19666007

[B49] Qu H.-X. Cui L. Meng X.-Y. Wang Z.-J. Cui Y.-X. Yu Y.-P. (2019). C1QTNF6 is overexpressed in gastric carcinoma and contributes to the proliferation and migration of gastric carcinoma cells. Int. J. Mol. Med. 43 (1), 621–629. 10.3892/ijmm.2018.3978 30431096

[B50] Qu L.-H. Hong X. Zhang Y. Cong X. Xiang R.-L. Mei M. (2021). C1q/tumor necrosis factor-related protein-6 attenuates TNF-α-induced apoptosis in salivary acinar cells via AMPK/SIRT1-modulated miR-34a-5p expression. J. Cell. Physiol. 236 (8), 5785–5800. 10.1002/jcp.30262 33400820

[B51] Rebouissou S. Nault J.-C. (2020). Advances in molecular classification and precision oncology in hepatocellular carcinoma. J. Hepatol. 72 (2), 215–229. 10.1016/j.jhep.2019.08.017 31954487

[B52] Schäffler A. Buechler C. (2012). CTRP family: Linking immunity to metabolism. Trends Endocrinol. Metab. 23 (4), 194–204. 10.1016/j.tem.2011.12.003 22261190

[B53] Seldin M. M. Tan S. Y. Wong G. W. (2014). Metabolic function of the CTRP family of hormones. Rev. Endocr. Metab. Disord. 15 (2), 111–123. 10.1007/s11154-013-9255-7 23963681 PMC3931758

[B54] Sequeira I. Rashid M. Tomás I. M. Williams M. J. Graham T. A. Adams D. J. (2020). Genomic landscape and clonal architecture of mouse oral squamous cell carcinomas dictate tumour ecology. Nat. Commun. 11 (1), 5671. 10.1038/s41467-020-19401-9 33168804 PMC7652942

[B55] Siegel R. L. Miller K. D. Fuchs H. E. Jemal A. (2021). Cancer statistics, 2021. Ca. Cancer J. Clin. 71 (1), 7–33. 10.3322/caac.21654 33433946

[B56] Smyth E. C. Nilsson M. Grabsch H. I. van Grieken N. C. Lordick F. (2020). Gastric cancer. Lancet (London, Engl. 396 (10251), 635–648. 10.1016/S0140-6736(20)31288-5 32861308

[B57] Song X. Li L. Shi L. Liu X. Qu X. Wei F. (2021). C1QTNF6 promotes oral squamous cell carcinoma by enhancing proliferation and inhibiting apoptosis. Cancer Cell Int. 21 (1), 666. 10.1186/s12935-021-02377-x 34906149 PMC8670214

[B58] Spyrou N. Avgerinos K. I. Mantzoros C. S. Dalamaga M. (2018). Classic and novel adipocytokines at the intersection of obesity and cancer: Diagnostic and therapeutic strategies. Curr. Obes. Rep. 7 (4), 260–275. 10.1007/s13679-018-0318-7 30145771

[B59] Sung H. Ferlay J. Siegel R. L. Laversanne M. Soerjomataram I. Jemal A. (2021). Global cancer statistics 2020: GLOBOCAN estimates of incidence and mortality worldwide for 36 cancers in 185 countries. Ca. Cancer J. Clin. 71 (3), 209–249. 10.3322/caac.21660 33538338

[B60] Takeuchi T. Adachi Y. Nagayama T. (2011). Expression of a secretory protein C1qTNF6, a C1qTNF family member, in hepatocellular carcinoma. Anal. Cell. Pathol. 34 (3), 113–121. 10.3233/ACP-2011-009 PMC4605662 21508531

[B61] Thanasupawat T. Glogowska A. Burg M. Krcek J. Beiko J. Pitz M. (2018). C1q/TNF-related peptide 8 (CTRP8) promotes temozolomide resistance in human glioblastoma. Mol. Oncol. 12 (9), 1464–1479. 10.1002/1878-0261.12349 29949238 PMC6120254

[B62] Tsimberidou A. M. Fountzilas E. Nikanjam M. Kurzrock R. (2020). Review of precision cancer medicine: Evolution of the treatment paradigm. Cancer Treat. Rev. 86, 102019. 10.1016/j.ctrv.2020.102019 32251926 PMC7272286

[B63] Vansaun M. N. (2013). Molecular pathways: Adiponectin and leptin signaling in cancer. Clin. Cancer Res. 19 (8), 1926–1932. 10.1158/1078-0432.CCR-12-0930 23355630 PMC3630242

[B64] Wan X. Zheng C. Dong L. (2019). Inhibition of CTRP6 prevented survival and migration in hepatocellular carcinoma through inactivating the AKT signaling pathway. J. Cell. Biochem. 120 (10), 17059–17066. 10.1002/jcb.28967 31111552

[B65] Wang L. Liu Z. Duan L. Ma B. Sun Z. (2015). C1q tumor necrosis factor-related protein 6 (CTRP6) inhibits the proliferation and migration of ovarian cancer cells. Chin. J. Cell. Mol. Immunol. 31 (12), 1664–1668. 26648301

[B66] Wang M. Tang X. Li L. Liu D. Liu H. Zheng H. (2018). C1q/TNF-related protein-6 is associated with insulin resistance and the development of diabetes in Chinese population. Acta Diabetol. 55 (12), 1221–1229. 10.1007/s00592-018-1203-2 30083983

[B67] Warnakulasuriya S. (2009). Global epidemiology of oral and oropharyngeal cancer. Oral Oncol. 45 (4-5), 309–316. 10.1016/j.oraloncology.2008.06.002 18804401

[B68] Wong G. W. Krawczyk S. A. Kitidis-Mitrokostas C. Revett T. Gimeno R. Lodish H. F. (2008). Molecular, biochemical and functional characterizations of C1q/TNF family members: Adipose-tissue-selective expression patterns, regulation by PPAR-gamma agonist, cysteine-mediated oligomerizations, combinatorial associations and metabolic functions. Biochem. J. 416 (2), 161–177. 10.1042/BJ20081240 18783346 PMC3936483

[B69] Wong G. W. Wang J. Hug C. Tsao T.-S. Lodish H. F. (2004). A family of Acrp30/adiponectin structural and functional paralogs. Proc. Natl. Acad. Sci. U. S. A. 101 (28), 10302–10307. 10.1073/pnas.0403760101 15231994 PMC478567

[B70] Wu W. Zhang J. Zhao C. Sun Y. Pang W. Yang G. (2017). CTRP6 regulates porcine adipocyte proliferation and differentiation by the AdipoR1/MAPK signaling pathway. J. Agric. Food Chem. 65 (27), 5512–5522. 10.1021/acs.jafc.7b00594 28535682

[B71] Wu Y. Zhang Y. Qin X. Geng H. Zuo D. Zhao Q. (2020). PI3K/AKT/mTOR pathway-related long non-coding RNAs: Roles and mechanisms in hepatocellular carcinoma. Pharmacol. Res. 160, 105195. 10.1016/j.phrs.2020.105195 32916254

[B72] Wullweber A. Strick R. Lange F. Sikic D. Taubert H. Wach S. (2021). Bladder tumor subtype commitment occurs in carcinoma *in situ* driven by key signaling pathways including ECM remodeling. Cancer Res. 81 (6), 1552–1566. 10.1158/0008-5472.CAN-20-2336 33472889

[B73] Xie Q. Chi S. Fang Y. Sun Y. Meng L. Ding J. (2021). PI3Kα inhibitor impairs AKT phosphorylation and synergizes with novel angiogenesis inhibitor AL3810 in human hepatocellular carcinoma. Signal Transduct. Target. Ther. 6 (1), 130. 10.1038/s41392-021-00522-6 33785733 PMC8010115

[B74] Xu E. Yin C. Yi X. Liu Y. (2020). Knockdown of CTRP6 inhibits high glucose-induced oxidative stress, inflammation and extracellular matrix accumulation in mesangial cells through regulating the Akt/NF-κB pathway. Clin. Exp. Pharmacol. Physiol. 47 (7), 1203–1211. 10.1111/1440-1681.13289 32077518

[B75] Xu F. Cui W.-Q. Wei Y. Cui J. Qiu J. Hu L.-L. (2018). Astragaloside IV inhibits lung cancer progression and metastasis by modulating macrophage polarization through AMPK signaling. J. Exp. Clin. Cancer Res. 37 (1), 207. 10.1186/s13046-018-0878-0 30157903 PMC6116548

[B76] Zanoni D. K. Montero P. H. Migliacci J. C. Shah J. P. Wong R. J. Ganly I. (2019). Survival outcomes after treatment of cancer of the oral cavity (1985-2015). Oral Oncol. 90, 115–121. 10.1016/j.oraloncology.2019.02.001 30846169 PMC6417804

[B77] Zhan S. Liu Z. Zhang M. Guo T. Quan Q. Huang L. (2019). Overexpression of B7-H3 in α-SMA-positive fibroblasts is associated with cancer progression and survival in gastric adenocarcinomas. Front. Oncol. 9, 1466. 10.3389/fonc.2019.01466 31998637 PMC6966326

[B78] Zhang H. E. Hamson E. J. Koczorowska M. M. Tholen S. Chowdhury S. Bailey C. G. (2019). Identification of novel natural substrates of fibroblast activation protein-alpha by differential degradomics and proteomics. Mol. Cell. Proteomics 18 (1), 65–85. 10.1074/mcp.RA118.001046 30257879 PMC6317473

[B79] Zhang J. Bai W.-P. (2022). C1q/tumor necrosis factor related protein 6 (CTRP6) regulates the phenotypes of high glucose-induced gestational trophoblast cells via peroxisome proliferator-activated receptor gamma (PPARγ) signaling. Bioengineered 13 (1), 206–216. 10.1080/21655979.2021.2012906 34964705 PMC8805812

[B80] Zhang J. He J. (2019). CTRP3 inhibits high glucose-induced oxidative stress and apoptosis in retinal pigment epithelial cells. Artif. Cells Nanomed. Biotechnol. 47 (1), 3758–3764. 10.1080/21691401.2019.1666864 31556307

[B81] Zhang W. Feng G. (2021). C1QTNF6 regulates cell proliferation and apoptosis of NSCLC *in vitro* and *in vivo* . Biosci. Rep. 41 (1), BSR20201541. 10.1042/BSR20201541 33269376 PMC7805025

[B82] Zhao Q. Zhang C.-L. Xiang R.-L. Wu L.-L. Li L. (2020a). CTRP15 derived from cardiac myocytes attenuates TGFβ1-induced fibrotic response in cardiac fibroblasts. Cardiovasc. Drugs Ther. 34 (5), 591–604. 10.1007/s10557-020-06970-6 32424654

[B83] Zhao Y. Zhang Y.-N. Wang K.-T. Chen L. (2020b). Lenvatinib for hepatocellular carcinoma: From preclinical mechanisms to anti-cancer therapy. Biochim. Biophys. Acta. Rev. Cancer 1874 (1), 188391. 10.1016/j.bbcan.2020.188391 32659252

[B84] Zheng W.-F. Zhang S.-Y. Ma H.-F. Chang X.-W. Wang H. (2019). C1qTNF-related protein-6 protects against doxorubicin-induced cardiac injury. J. Cell. Biochem. 120 (6), 10748–10755. 10.1002/jcb.28366 30719766

[B85] Zheng Y. Wang J. Zhao T. Wang L. Wang J. (2021). Modulation of the VEGF/AKT/eNOS signaling pathway to regulate liver angiogenesis to explore the anti-hepatic fibrosis mechanism of curcumol. J. Ethnopharmacol. 280, 114480. 10.1016/j.jep.2021.114480 34358654

[B86] Zhu X. Tong H. Gao S. Yin H. Zhu G. Li X. (2020). C1QTNF6 overexpression acts as a predictor of poor prognosis in bladder cancer patients. Biomed. Res. Int. 2020, 7139721. 10.1155/2020/7139721 33123583 PMC7585664

[B87] Zou J. Wang E. (2019). Cancer biomarker discovery for precision medicine: New progress. Curr. Med. Chem. 26 (42), 7655–7671. 10.2174/0929867325666180718164712 30027846

